# Taxonomic response of bacterial and fungal populations to biofertilizers applied to soil or substrate in greenhouse-grown cucumber

**DOI:** 10.1038/s41598-022-22673-4

**Published:** 2022-11-02

**Authors:** Jiajia Wu, Zhaoai Shi, Jiahong Zhu, Aocheng Cao, Wensheng Fang, Dongdong Yan, Qiuxia Wang, Yuan Li

**Affiliations:** 1 Institute of Plant Protection, Chinese Academy of Agricultural Sciences, Beijing, 100193 China; 2 Beijing Innovation Consortium of Agriculture Research System, Beijing, 100193 China

**Keywords:** Ecology, Environmental sciences

## Abstract

Reductions in the quality and yield of crops continuously produced in the same location for many years due to annual increases in soil-borne pathogens. Environmentally-friendly methods are needed to produce vegetables sustainably and cost effectively under protective cover. We investigated the impact of biofertilizers on cucumber growth and yield, and changes to populations of soil microorganisms in response to biofertilizer treatments applied to substrate or soil. We observed that some biofertilizers significantly increased cucumber growth and decreased soil-borne pathogens in soil and substrate. Rhizosphere microbial communities in soil and substrate responded differently to different biofertilizers, which also led to significant differences in microbial diversity and taxonomic structure at different times in the growing season. Biofertilizers increase the prospects of re-using substrate for continuously producing high-quality crops cost-effectively from the same soil each year while at the same time controlling soil-borne disease.

## Introduction

Cucumber is an important vegetable crop that is extensively grown and consumed worldwide. Almost 2 Mha were used worldwide to produce about 75 Mt of cucumber in 2018^1^. China is one of the main countries that produces cucumber. The total cucumber production in China in 2018 was about 56 Mt on about 1 Mha, equivalent to about 53 and 75% of the global quantity and area, respectively^2^.

Soil-borne diseases such as *Fusarium wilt* and root-knot nematodes (RKN, *Meloidogyne* spp.) significantly decrease cucumber yield and quality by accumulating in the same soil used to produce crops every year^3,4^. Those pathogens when left uncontrolled can affect large production areas and thereby significantly limit cucumber production, quality, and expansion of the industry.

Soil microorganisms play an indispensable role in the growth and development of plants. They are crucial for the decomposition and transformation of organic matter in the soil, including the degradation of animal and plant residues, the decomposition of humus and the recycling and utilization of nutrients, and even the degradation and purification of organic pollutants^5,6^. The growth and quality of cucumbers can also be due to the relative abundance of rhizosphere microorganisms^7^. Plant disease has been correlated with changes in the soil microecology that favors plant pathogens^8^.

Excessive dependence on chemical fertilizers to increase crop yield in industrial-scale agricultural production has damaged the environment and harmed human health^9^. Chemical fertilizers can replace biofertilizers which contain beneficial microorganisms that can enhance crop production without harm to human health^10,11^.

Nutrients and beneficial microflora contained in biofertilizers have led to improved crop production and quality^8,12^. They have been reported to reduce ecological degradation and suppress soil-borne diseases^13^. Beneficial microorganisms such as *Trichoderma harzianum* and *Bacillus subtilis* in biofertilizers promoted colonization by other microbes, inhibited pathogens, and induced plant systemic resistance to disease^14^.

*Trichoderma* is a commercially-available biocontrol fungal agent applied to soil to control soil-borne pathogens and improve crop growth. *Trichoderma* exhibits several useful properties including mycoparasitism^15^, absorption of nutrients, good colonization ability^16^, production of antibiotic substances and promotion of plant systemic resistance to pathogens^17,18^. *Trichoderma* stimulated the yield and promoted the establishment of communities of beneficial soil microflora, thereby improving soil microbial activity and enhancing soil fertility^19^.

*Bacillus* is very common bacterium that significantly promoted plant growth and crop yield^11^. *Bacillus* stimulated synthesis of auxin in plants, and formed endospores and different biologically active compounds important for the biocontrol of plant pathogens^20,21^.

Substrates can reduce pathogens accumulating in soil-based crops that are continually produced in the same location for many years^22^. Many substrates used in commercial production are not recycled and some were obtained from non-renewable resources, both of which are inconsistent with best-practice sustainable crop production. There is therefore an urgent need to find and implement substrates from renewable resources that are recyclable.

Rhizosphere microorganisms present in substrates were not only the driving force for organic nutrient transformation^23^, but also their structure and abundance affected plant growth and development^24^. Exploring the effects of exogenous microbial agents on cucumber yield, quality and rhizosphere environment in substrates has important theoretical and practical significance for improving substrate production and their use.

We found no reports of microbial taxonomic changes in response to biofertilizers added to soil or substrates. Our research therefore aimed to: (1) Determine and compare the effects of different microbial fertilizers on the growth of cucumber plants grown in soil or substrate; (2) Determine the effect on the taxonomic structure and function of bacterial and fungal populations exposed to different microbial fertilizers continually applied to soil or substrates; (3) Determine and compare the responses of soil-borne pathogens exposed to different microbial fertilizers in soil or substrates; and (4) Determine cucumber yield in response cucumber plants grown in soil or substrates exposed to different microbial fertilizers.

## Materials and methods

### Greenhouse location

In 2021, a large greenhouse in the Changping district of Beijing (GPS: 40° 22′ N, 116° 23′ E) was selected for the research trials. Cucumbers had been continually produced in the greenhouse since 2016. The main components of the substrate include: peat, coconut bran, perlite, vermiculite, and rice husk. The basic physicochemical characteristics of the soil and substrate were shown in Table S1.

### Biofertilizers

#### Preparation of *Trichoderma* spore suspension

*Trichoderma afroharzianum* 267 strain (‘Strain 267’) had been identified and stored in our laboratory since 2019. Strain 267 was cultured on PDA for 5 days, and then the spore suspension was washed with sterilized and distilled water before being filtered through 4-layer gauze. The concentration of the spore suspension was adjusted to 1.0 ± 0.05 × 10^7^ spores/mL using a hemocytometer.

#### Commercial microbial fertilizers

*Bacillus subtilis* (BS) or *T. harzianum* (HZ) biofertilizers were sourced from Hainan Jin Yufeng Biological Engineering Co., Ltd., China.

A compound microbial fertilizer (M) with several kinds of functional bacteria was sourced from Inner Mongolia Shengtian Agricultural Technology Co., Ltd. (Mongolia).

### Field experiment design

Cucumber seedlings grown from seed (Jingyou 4, Beijing Wanlongyufeng Seed Co., Ltd., China) were transplanted to soil in the greenhouse in early May 2021. The area of each plot was 1.2 m wide × 25 m long. Six biofertilizer treatments in soil (S) were coded as: Strain 267 (S267), *B. subtilis* (SBS), *B. subtilis*/*T*. *harzianum* (SBH), compound microbial fertilizer (SM), *T*. *harzianum* (SHZ) and control (SCK). Six treatments in substrate (US) were similarly coded: US267, USBS, USBH, USM, USHZ, USCK.

The 12 treatments were diluted with deionized water and established in plots following a random block design. The biofertilizers were applied 2, 3 or 4 weeks after the cucumber seedlings were planted in the greenhouse.

Cucumber roots were treated with 50 mL/plant of *Trichoderma* spore suspension (1.0 ± 0.05 × 10^7^ spores/mL, treatment 1: Strain 267). Four further commercial biofertilizer treatments were applied to the cucumber seedling roots that comprised 72 mL of *B. subtilis* biofertilizer diluted 400 times (Treatment 2: BS); 36 mL of *B. subtilis* and 20 g *T. harzianum* biofertilizer diluted 400 times (Treatment 3: BH); 72 mL of compound microbial fertilizer diluted 400 times (Treatment 4: M); 40 g of *T. harzianum* biofertilizer diluted 400 times (Treatment 5: HZ). A sixth treatment was the control consisting of deionized water added to soil and substrate (Treatment 6: CK). Each treatment was replicated 3 times with 100 cucumber seedlings in each replicate.

### Sample collection

Soil and substrate were sampled from each treatment 2–20 cm deep on day 7 after the third application of biofertilizer and when the cucumber plants were uprooted. Soil samples were refrigerated at − 80 and 4 ℃ for later analysis of taxonomic changes in the microbial community and soil-borne pathogens.

### Soil and substrate physicochemical properties

A Futura Continuous Flow Analytical System (Alliance Instruments, France) was used to quantify ammonia nitrogen (NH_4_^+^–N) and nitrate nitrogen (NO^3–^N) concentrations in each soil sample. The available phosphorus (P) was determined according to the method described by Olsen et al.^25^. Available potassium (K) was determined using a FP640 Flame Photometer (Shanghai Instruments Group Co., Ltd., China). The organic matter (OM) content was quantified according to the K_2_Cr_2_O_7_–H_2_SO_4_ oxidation reduction method described by Schinner et al.^26^. A MP512-02 Precision Water Meter was used to measure the pH of the soil sample (Shanghai Sanxin Instrumentation, Inc., China). A MP513 Conductivity Meter was used to determine the electrical conductivity (EC) (Shanghai Sanxin Instrumentation, Inc., China) of the soil.

### Cucumber growth, yield, and soil-borne pathogens

Cucumber height and stem diameter were measured every 7 days from the day the seedlings were transplanted. The total marketable yield of cucumber from each treatment was recorded in kg at each harvest.

Selective medium methods were used to isolate colonies of *Fusarium* spp. and *Phytophthora* spp. in the soil, and to calculate their abundance following the methods described by Komada^27^ and Masago^28^, respectively.

### Extraction of soil and substrate DNA, and PCR amplification

Ten grams of soil or substrate were homogenized in 40 mL of sterile water. The homogenate was then filtered through two layers of sterile medical gauze. The gauze was rinsed several times with sterile water to recover residual microorganisms. The filtrate was centrifuged at 10,000–12,000*g* at 4 ℃ for 15 min.

The total soil and substrate DNA was extracted from each 0.25 g soil and substrate sample following the procedures described in the DNeasy Power Soil Pro Kit (Qiagen Com., China). The extracted DNA was plated out onto 1% agarose gel for electrophoresis, and then the DNA concentration measured using a NanoDrop ND-1000 UV–Vis Spectrophotometer (Thermo Fisher Scientific Inc., USA).

The bacterial universal primers 338F [5′-ACTCCTACGGAGCAGGCAG-3′] and 806R [5′-GGACTACHGGGGTWTCTAAT-3′]^29^, and the fungal universal primers ITS1F [5′-CTTGGTCATAGAGGAGTAA-3′] and ITS2R [5′-GCTGCTATCGATGC-3′]^30^, were used to amplify the V3-V4 region of bacteria and the ITS1-ITS2 region of fungi, respectively. PCR products were detected by gel electrophoresis (plated out on 2% agarose) and purified using the EasyPure Quick Gel Extraction Kit (TransGen Biotech Co. Ltd., China) and quantified using the QuantiFluor dsDNA System (Fisher Scientific, USA).

### High-throughput sequencing

The purified PCR products were sequenced at Majorbio Bio-Pharm Technology Co. Ltd. (Shanghai, China) and the microbial analyses were conducted using the MiSeq PE300 sequencing platform (Illumina Com., USA).

The raw sequences were processed using the Mothur software (Version 1.30.2 https://www.mothur.org/wiki/Download_mothur). Sequences with less than 50 bp, ambiguous bases and those with an average mass less than 20 were removed by FLASH (Version 1.2.11 https://ccb.jhu.edu/software/FLASH/index.shtml) and Trimmomatic (Version 0.39 http://www.usadellab.org/cms/?page=trimmomatic) software to obtain the effective sequences^31^.

Usearch (Version 7.1 http://drive5.com/uparse/) software was used to cluster sequences with 97% similarity into Operational Taxonomic Units (OTUs). Qiime software (Version1.9.1 http://qiime.org/install/index.html) and Unit database (V7.2 https://unite.ut.ee/) were used for species annotation analysis and sample community composition analysis^32^.

Qiime software (Version 1.9.1) was used to calculate the richness of the flora (Chao1 index, Shannon index) and the diversity of the flora (Simpson index, Ace index). R software (Version 2.15.3 https://cran.r-project.org/doc/FAQ/R-FAQ.html#Citing-R) was used to draw the dilution curve and bar diagrams of species at genus level^33^.

### Statistical analysis

The efficacy of the treatments on soil-borne pathogens was calculated using this formula:

$${\text{Y}} = \frac{{X_{0} - X_{1} }}{{X_{0} }} \times 100$$

where Y is the relative efficacy on soil-borne pathogens (%), X_0_ is the colony number of soil-borne pathogens in the control group, and X_1_ is the colony number of soil-borne pathogens in treatment group.

The data were analyzed as a one-way ANOVA using the IBM SPSS Statistics 25 software package (IBM, USA). Before the one-way ANOVA, we performed the Normal distribution test and the homogeneity of variance test (F-test) with the IBM SPSS Statistics 25 software package (IBM, USA), and the raw data met the conditions of ANOVA. Significant differences between treatments were identified using Duncan's new multiple range test at the 0.05 level of significance. All treatments were compared with the control, except where specifically stated.

### A statement on guidelines

Plants experiments in our study complies with the the People's Republic of China plant Bank guideline and legislation.

## Results

All the results were reported relative to the control, unless specifically stated to the contrary or for clarity.

### Growth of cucumber plants in response to different biofertilizers

#### Soil

**Figure 1:**
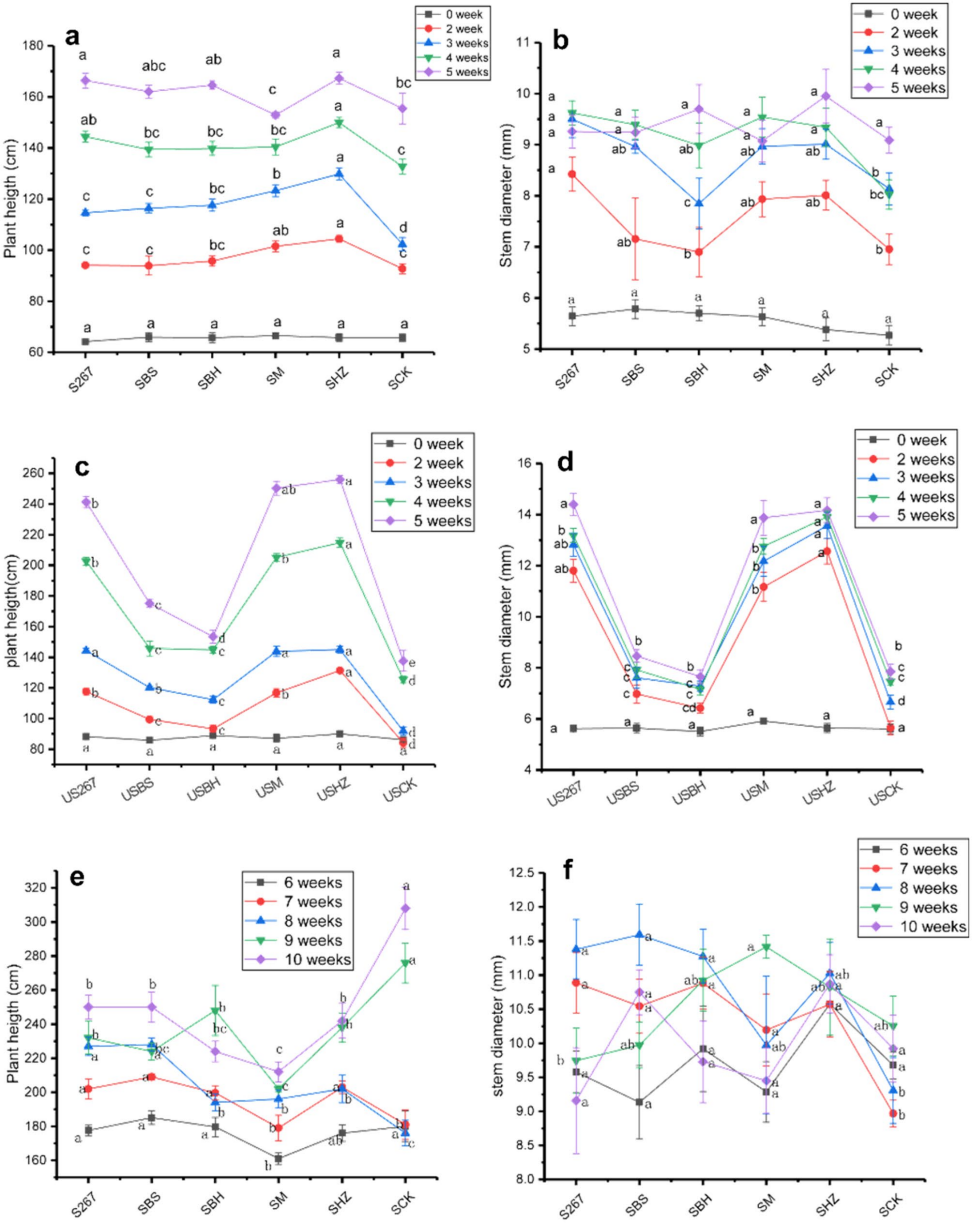

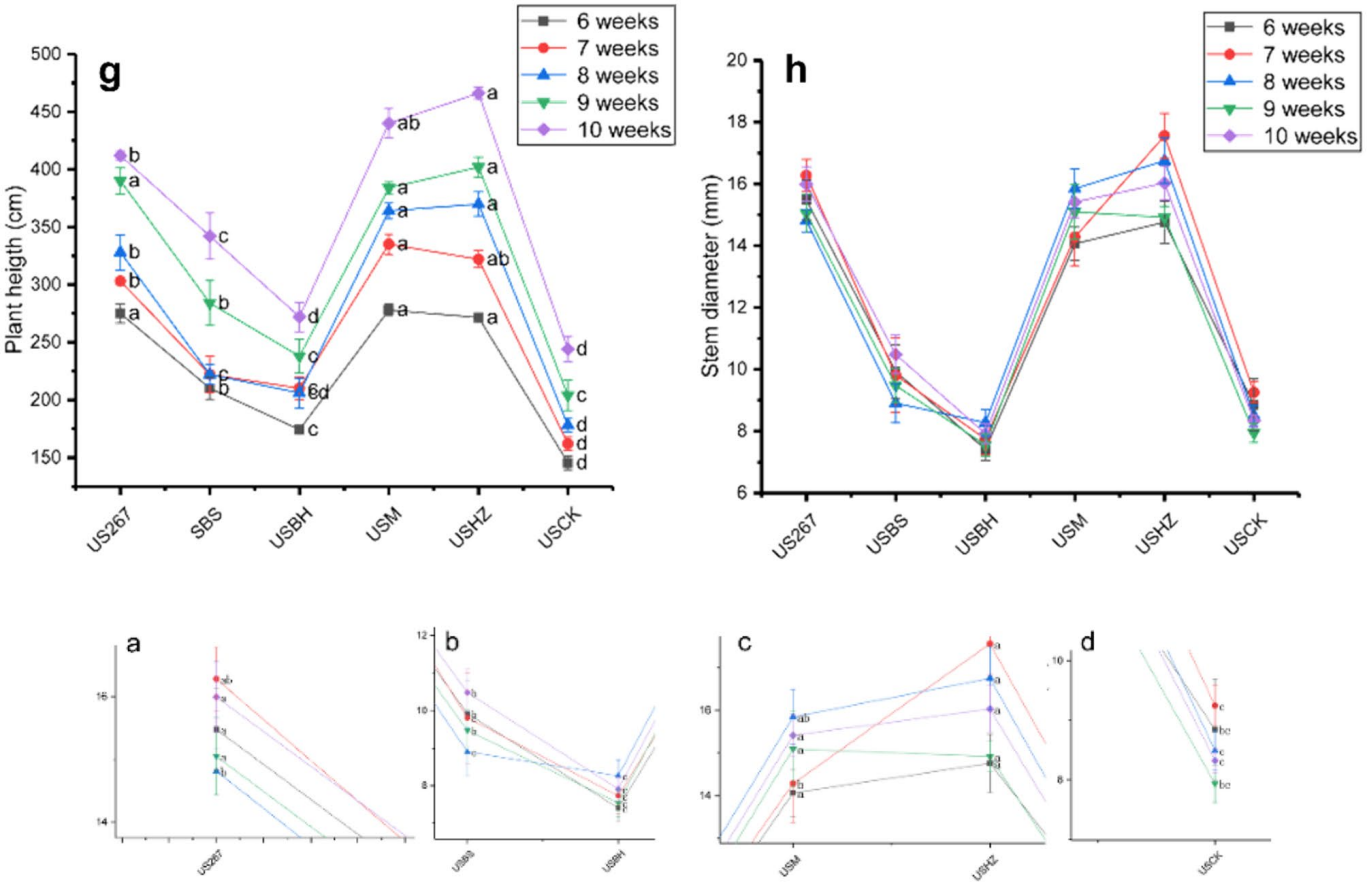
Effect of different biofertilizer treatments on the growth of cucumber seedlings produced in soil or substrate in a greenhouse. S267 = *Trichoderma Strain 2*67 added to soil; SBH = *Bacillus subtilis* and *T. harzianum* biofertilizers added to soil; SBS = *B. subtilis* biofertilizer added to the soil; SM = Compound biofertilizer added to soil; SHZ = *T. harzianum* biofertilizer added to soil; SCK = Untreated soil. US267 = *T.*267 biofertilizer added to substrate; USBH = *B. subtilis* and *T. harzianum* biofertilizers added to substrate; USBS = *B. subtilis* biofertilizer added to substrate; USM = Compound biofertilizer added to substrate; USHZ = *T. harzianum* biofertilizer added to substrate; USCK = Untreated substrate.

There was no significant difference in cucumber growth before microbial fertilizer was applied. However, some microbial fertilizers significantly increased cucumber height and stem diameter when they were applied within 4 weeks from when the seedlings were planted (Fig. 1a,b,e,f). In the second week, SHZ and SMF increased plant height by 11.2 and 9.5%, respectively. In the third week, S267, SBS, SBH, SM and SHZ increased plant height by 12.0, 13.8, 15.0, 20.5 and 26.9%, respectively (Fig. 1a). In the fourth and fifth weeks, some treatments significantly increased cucumber height. In the second and third weeks, S267 significantly increased stem diameter by 21.2 and 16.8% (Fig. 1b).

Over the subsequent 5 weeks, some microbial fertilizer treatments decreased cucumber height and stem diameter (Fig. 1g,h).

#### Substrate

There were no significant differences in cucumber growth before microbial fertilizer microbial fertilizer was applied (Fig. 1c,d,g,h). However, within 4 weeks of applying the microbial fertilizer, each biofertilizer treatment applied significantly increased cucumber height (Fig. 1c). US267 and USHZ significantly increased cucumber height by 39.8–75.4% and 56.1–86.1%, respectively. US267, USM and USHZ significantly increased the stem diameter by 76.8–108.9%, 71.1–97.6% and 80.4–122.4%, respectively (Fig. 1d).

Over the subsequent 5 weeks, US267, USM and USHZ treatments continued to significantly increase cucumber height and stem diameter (Fig. 1g,h).

### Changes in the taxonomic composition of soil-borne fungal pathogens

#### Soil

Biofertilizers application significantly reduced the taxonomic composition of soil-borne fungal pathogens at different times during the cucumber growth period (Tables 1 and 2). *Fusarium* spp. were significantly reduced (T, 63.8% reduction, P < 0.001) after the third application of *T. harzianum*, which was during the early period ofgrowth (Table 1). *Phytophthora* spp. was significantly reduced (T, 81.6% reduction, P < 0.001) by Strain 267. *Fusarium* spp. and *Phytophthora* spp. were significantly reduced by S267, SBS and SHZ. When the cucumber were uprooted during the later period of growth, we observed that SBS, SHZ and S267 treatments had significantly reduced *Fusarium* spp. and *Phytophthora* spp. Therefore, S267, SBS and SHZ treatments were considered as the most effective biofertilizers against *Fusarium* spp. or *Phytophthora* spp.

**Table 1:** Average number of *Fusarium* spp. and *Phytophthora* spp. in soil, and the percent relative efficacy of biofertilizers in the early and late stages of cucumber growth.

Treatment	Early-stage		Late-stage	
	*Fusarium* spp.	*Phytophthora* spp.	*Fusarium* spp.	*Phytophthora* spp.
S267	46.54 ± 2.39ab	81.57 ± 2.14a	30.66 ± 3.49b	61.47 ± 2.04a
SBS	62.95 ± 7.60a	55.79 ± 1.31b	61.31 ± 1.06a	33.59 ± 0.64b
SBH	41.07 ± 4.31b	29.83 ± 2.98c	13.87 ± 1.01d	16.09 ± 2.33c
SM	19.09 ± 5.32c	15.62 ± 5.97d	22.47 ± 2.55c	33.89 ± 0.20b
SHZ	63.84 ± 8.22a	61.01 ± 4.57b	56.69 ± 2.85a	56.97 ± 0.39a
SCK	11,947	11,867	8220	13,633

Means (N = 3) within the same time period accompanied by the same letter are not statistically different (P = 0.05), according to Duncan's new Multiple-Range test. S267 = *Trichoderma Strain 2*67 added to soil; SBH = *Bacillus subtilis* and *T. harzianum* biofertilizers added to soil; SBS = *B. subtilis* biofertilizer added to the soil; SM = Compound biofertilizer added to soil; and SHZ = *T. harzianum* biofertilizer added to soil.

**Table 2:** Average number of *Fusarium* spp. and *Phytophthora* spp. in substrate and percent relative efficacy of biofertilizers in the early and late stages of cucumber growth.

Treatment	Early-stage		Late-stage	
	*Fusarium* spp.	*Phytophthora* spp.	*Fusarium* spp.	*Phytophthora* spp.
US267	50.20 ± 2.19b	66.79 ± 2.66a	66.54 ± 0.65c	47.97 ± 0a
USBS	87.49 ± 1.33a	57.02 ± 4.44ab	95.39 ± 0.09a	33.53 ± 1.28b
USBH	22.05 ± 9.51c	46.40 ± 2.64bc	96.64 ± 1.57a	40.89 ± 2.36ab
USM	26.80 ± 2.61c	37.85 ± 7.82c	61.88 ± 0.57d	36.63 ± 6.77b
USHZ	52.83 ± 4.62b	61.23 ± 3.71ab	84.01 ± 1.92b	33.24 ± 0.09b
USCK	6867	18,707	36,480	13,760

Means (N = 3) within the same time period accompanied by the same letter are not statistically different (P = 0.05), according to Duncan's new Multiple-Range test. US267 = *T.*267 biofertilizer added to substrate; USBH = *B. subtilis* and *T. harzianum* biofertilizers added to substrate; USBS = *B. subtilis* biofertilizer added to substrate; USM = Compound biofertilizer added to substrate; USHZ = *T. harzianum* biofertilizer added to substrate; USCK = Untreated substrate.

Strain 267, BS, and HZ effectively controlled soil-borne pathogens. However, the effect of *T.*267 and HZ decreased during the late growth stage of cucumber.

#### Substrate

*Fusarium* spp. was significantly reduced (T, 87.5% reduction, P < 0.001) after the third application of USBS, which was during the early period of growth (Table 2). *Phytophthora* spp. was significantly reduced (T, 81.6% reduction, P < 0.001) by strain 267. *Fusarium* spp. and *Phytophthora* spp. were significantly reduced by US267, USBS and USHZ. At the time the cucumberwere uprooted during the later period of growth, each treatment had significantly reduced *Fusarium* spp. by more than 60%. In addition, *Phytophthora* spp. was significantly reduced (T, 47.9% reduction, P < 0.001) by US267. Therefore, US267, USBS and USHZ treatments were considered as the most effective treatments against *Fusarium* spp. and *Phytophthora* spp.

Strain 267, BS, and HZ effectively controlled soil-borne pathogens. The effect of *T.*267 and HZ increased during the late growth stage of cucumber.

### Changes in cucumber yield

**Figure 2:**
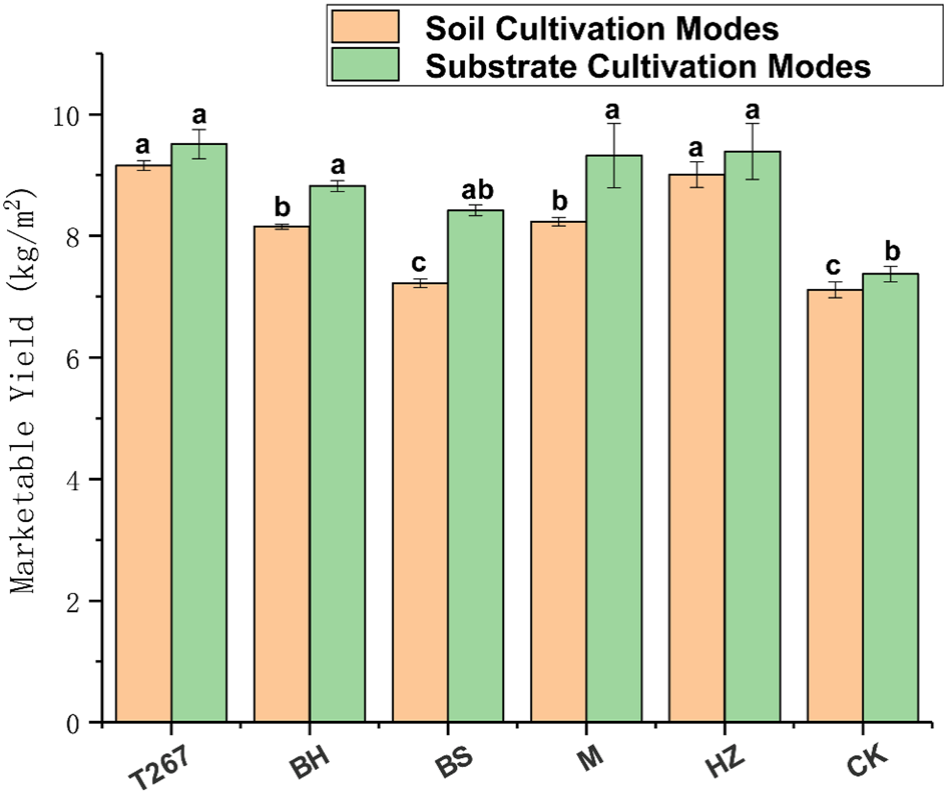
Effect of different biofertilizer treatments on the total marketable yield of cucumber. Means (N = 3) within the same time period accompanied by the same letter were not statistically different (P = 0.05), according to Duncan's new Multiple-Range test. S267 = *Trichoderma Strain 2*67 added to soil; SBH = *Bacillus subtilis* and *T. harzianum* biofertilizers added to soil; SBS = *B. subtilis* biofertilizer added to the soil; SM = Compound biofertilizer added to soil; SHZ = *T. harzianum* biofertilizer added to soil; SCK = Untreated soil. US267 = *T.*267 biofertilizer added to substrate; USBH = *B. subtilis* and *T. harzianum* biofertilizers added to substrate; USBS = *B. subtilis* biofertilizer added to substrate; USM = Compound biofertilizer added to substrate; USHZ = *T. harzianum* biofertilizer added to substrate; USCK = Untreated substrate.

Some treatments significantly increased cucumber yield after microbial fertilizer applications to plants grown in soil (Fig. 2). S267, SBS, SM and SHZ significantly increased cucumber yield. S267 and SHZ significantly increased cucumber yield by 28.8 and 26.7%, respectively.

US267, USM and USHZ significantly increased cucumber yield by 29.0, 26.5 and 27.4% when they were applied to seedlings grown on substrate (Fig. 2).

### Taxonomic changes to bacterial and fungal populations in soil or substrate

#### Diversity analysis of bacteria

A total of 1,709,972 valid reads were obtained from 16S rRNA amplicon sequencing after trimming. The average length of the amplicon was 428 bp. The number of valid sequences detected for each soil sample exceeded 40,000. The sparse curve was flat, which indicated that the genetic data were sufficient for a reasonable estimate of the total taxonomic composition.

Analysis of the microbial community diversity index in soil in response to the each biofertilizer treatment showed that the OTU diversity and richness were like the control (Fig. S1a,b,e,f). In the early stage of the cucumber growth period and after the third application of biofertilizer, and in the late stage of the cucumber growth period when the cucumber were uprooted, there were significant changes observed in the soil and substrate α diversity in the microbial community.

In the early stage of cucumber growth, the Shannon diversity index in SBS increased significantly (Fig. S1a). However, in the late stage of cucumber growth, there was no significant difference in the Shannon diversity index (Fig. S1e). The Chao1 richness index increased significantly in SM (Fig. S1f).

Analysis of the microbial community diversity index in substrate in response to each biofertilizer treatment showed that the OTU diversity and richness were significantly different to the control (Fig. S1c,d,g,h).

In the early stage of cucumber growth, the Shannon diversity index decreased significantly in USBS, USBH and USHZ (Fig. S1c). However, the Shannon diversity index increased significantly in US267 and USM.

The Chao richness index decreased significantly in USBH, USHZ and US267 (Fig. S1d). In the late stage of cucumber growth, the Shannon diversity index decreased significantly in USM (Fig. S1g). The Chao richness index increased significantly in USHZ and US267 (Fig. S1h).

#### Bacterial hierarchical cluster analysis at the OTU level

The results of hierarchical cluster analysis showed that different treatments had different effects on the taxonomic structure of microbes in the soil or substrate at different stages of cucumber plant growth (Fig. S3a–d).

In the early stage of cucumber growth, the microbial communities exposed to SM and in the control were genetically close, compared with the other biofertilizer treatments, which indicated that the soil microbial community structure was changed more by the other biofertilizer treatments than SM (Fig. S3a). However, in the later stage of cucumber growth the five biofertilizer treatments were well-separated, which indicated that the microbial community structure was changed by the treatments (Fig. S3c).

In the early stage of cucumber growth in substrates, the control bacterial taxa were well-separated from the five biofertilizer treatments (Fig. S3b). However, the five treatments and control were observed divided into two branches, one for US267 and the other for USCK, USBH, USBS, USM and USHZ. The structure of the microbial community was similar in USBH, USBS, USM and USHZ and the control. The results indicated that US267 had significantly changed the taxonomic structure of the bacterial community.

In the late stage of cucumber growth, the five biofertilizer treatments and the control were observed as divided into two branches, one for USCK, USBS and USBH and the other for US267, USM and USHZ (Fig. S3d). The taxonomic structure of the microbial community was similar in the USBH and USBS to the control. The results indicated that US267, USM and USHZ had significantly changed the composition of the bacterial taxa.

#### Changes in bacterial genera dominance

**Figure 3:**
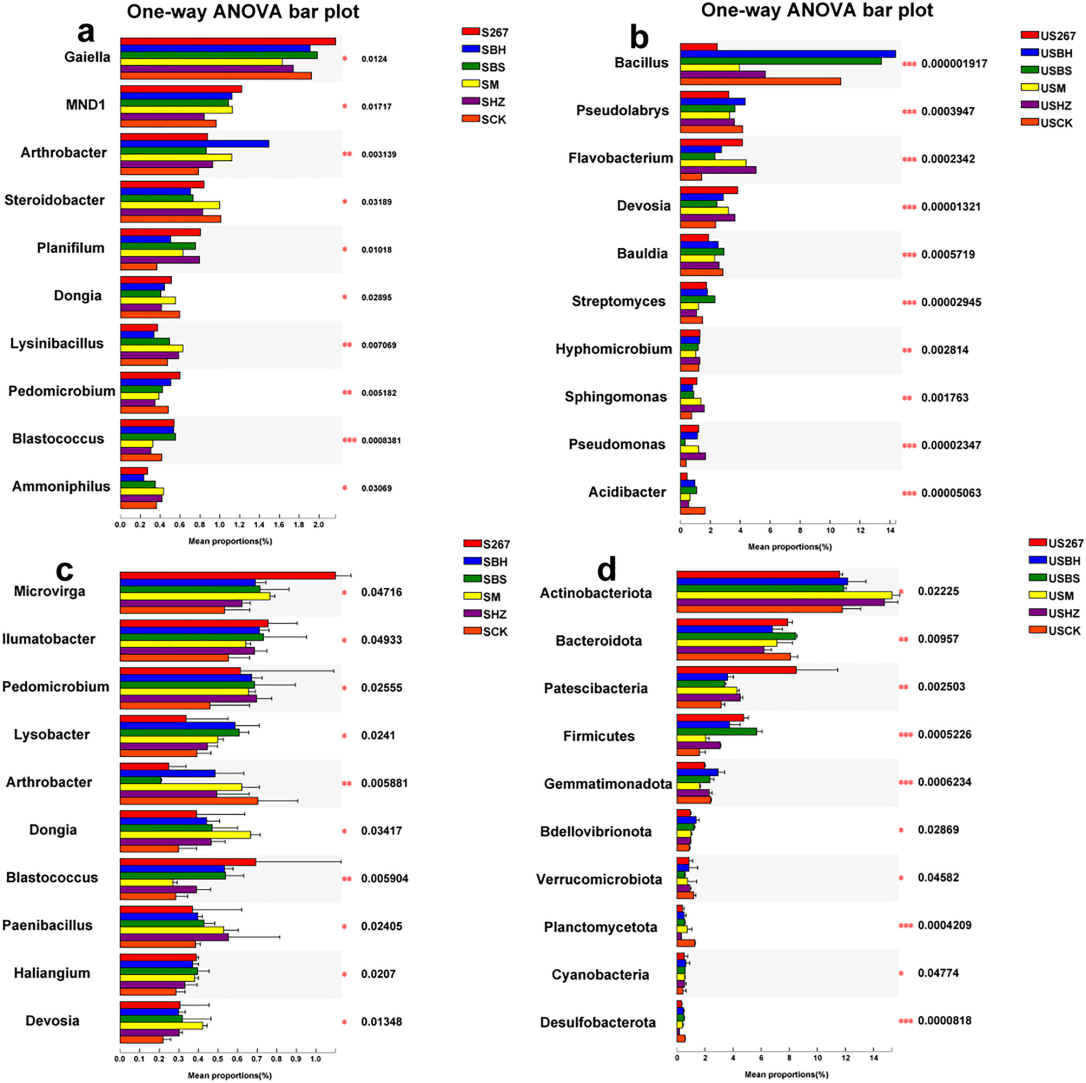
Relative abundance of bacterial genera detected in soil (**a** early samples; **c** late samples) and substrate (**b** early samples; **d** late samples) after different biofertilizer treatments. The number of asterisks indicates significant differences between treatments according to a one-way ANOVA and FDR (False Discovery Rate) adjustment (*0.01 < P ≤ 0.05; **0.001 < P ≤ 0.01; ***P ≤ 0.001). S267 = *Trichoderma Strain 2*67 added to soil; SBH = *Bacillus subtilis* and *T. harzianum* biofertilizers added to soil; SBS = *B. subtilis* biofertilizer added to the soil; SM = Compound biofertilizer added to soil; SHZ = *T. harzianum* biofertilizer added to soil; SCK = Untreated soil. US267 = *T.*267 biofertilizer added to substrate; USBH = *B. subtilis* and *T. harzianum* biofertilizers added to substrate; USBS = *B. subtilis* biofertilizer added to substrate; USM = Compound biofertilizer added to substrate; USHZ = *T. harzianum* biofertilizer added to substrate; USCK = Untreated substrate.

In the early stage of cucumber growth, significant differences were observed in the relative abundance of bacterial genera after applying microbial fertilizer (Fig. 3a). *MND1* was second only to *Gaiella* in dominance. The relative abundance of *Planifilum* also increased significantly at that time. In the late stage of cucumber growth, *Ilumatobacter* became second only to *Microvirga* in dominance (Fig. 3c). The relative abundance of *Arthrobacter, Pedomicrobium, Dongia, Haliangium,* and *Devosia* also increased significantly at that time.

In the early stage of cucumber growth, *Pseudolabrys* was second only to *Bacillus* in dominance (Fig. 3b). The relative abundance of *Flavobacterium, Sphingomonas* and *Pseudomonas* also increased significantly. In the late stage of cucumber growth, *Bacteroidota* became second only to *Actinobacteriota* in dominance (Fig. 3d). *Actinobacteriota* increased significantly in USM and USHZ at that time. *Bacteroidota* decreased significantly in USBH and USHZ. The relative abundance of *Firmicutes* increased significantly.

**Figure 4:**
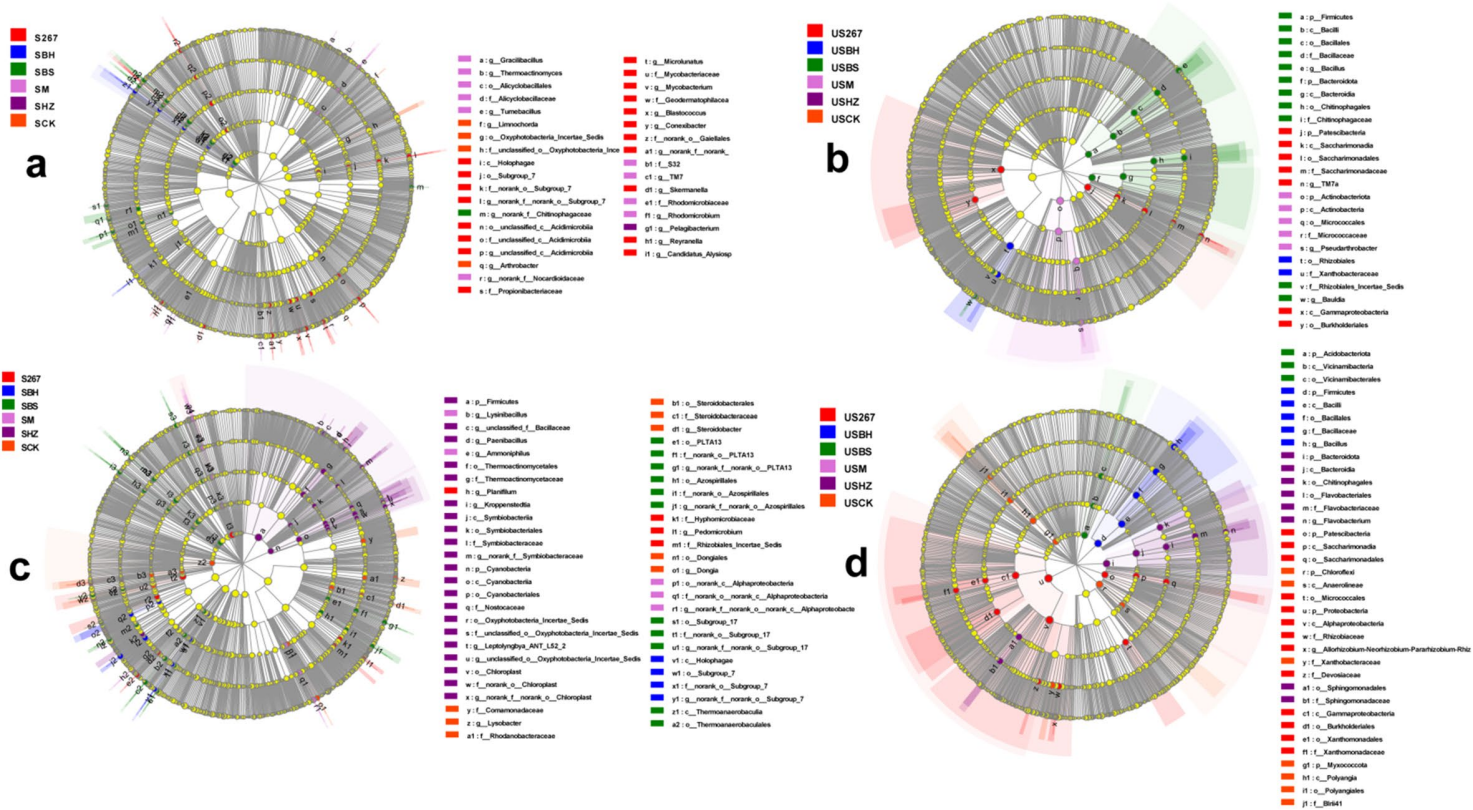
LEfSe cladogram analysis of species differential abundance in soil (**a** early samples; **c** late samples) and substrate (**b** early samples; **d** late samples) in bacterial communities after different biofertilizer treatments. S267 = *Trichoderma Strain 2*67 added to soil; SBH = *Bacillus subtilis* and *T. harzianum* biofertilizers added to soil; SBS = *B. subtilis* biofertilizer added to the soil; SM = Compound biofertilizer added to soil; SHZ = *T. harzianum* biofertilizer added to soil; SCK = Untreated soil. US267 = *T.*267 biofertilizer added to substrate; USBH = *B. subtilis* and *T. harzianum* biofertilizers added to substrate; USBS = *B. subtilis* biofertilizer added to substrate; USM = Compound biofertilizer added to substrate; USHZ = *T. harzianum* biofertilizer added to substrate; USCK = Untreated substrate.

LEfSe analysis identified biomarkers that caused significant differences in control (P < 0.05, a, c, LDA = 3; b, d, LDA = 4.0; Fig. 4a,b,c,d). Fig. 4a,b,c,d showed five rings in the cladogram, from inside to outside, representing the phylum, class, order, family, and genus taxonomic levels, respectively. The different color nodes (except yellow) on the ring represent significant changes in taxonomic composition due to the biofertilizer treatments.

The non-parametric factorial Kruskal–Wallis (KW) sum-rank test and Linear Discriminant Analysis (LDA) estimated the magnitude of the effect of each component (species) abundance on the differential effect. We observed that at the bacterial genus level some major taxa were screened out, which suggested that these biomarkers have the greatest impact on the results. In the early stage of cucumber growth in soil, 3, 1 and 1 biomarkers were found in S267, SBS and SCK, respectively. In the late stage of cucumber growth, 3, 3, 1, 3, 3 and 3 biomarkers were found in S267, SBS, SBH, SM, SHZ and SCK, respectively. In the early stage of cucumber growth in substrate, 1, 2 and 1 biomarkers were found in US267, USBS and USM, respectively. In the late stage of cucumber growth, 1, 1 and 1 biomarkers were found in US267, USBH and USHZ, respectively.

The results indicated that g__norank_f__norank_o__*Gaiellales* and g__*Blastococcus* became prevalent when S267 was applied to the soil during the early stage of cucumber growth, and that g__*Paenibacillus* became prevalent when SHZ was applied at the late stage of cucumber growth.

LEfSe analysis confirmed that USBS significantly increased the abundance of the c__*Bacilli* at the early stage of cucumber growth in substrate, and that US267 and USHZ significantly increased the abundance of p__*Patescibacteria* and p__*Firmicutes*, respectively, at the late stage of cucumber growth.

#### Changes in fungal genera dominance

A total of 1,859,284 valid reads were obtained from ITS rRNA amplicon sequencing after quality trimming. The average length of the amplicon was 321 bp. The number of valid sequences detected for each soil sample exceeded 40,000. The sparse curve was flat which indicated that the genetic data were sufficient for a reasonable estimate of the total taxonomic composition.

We observed that the microbial community α diversity of soil in the early and late stages of cucumber growth changed significantly (Fig. S2a,b,e,f). In the early stage of cucumber growth, there were no significant differences in the diversity index of Shannon and the richness index of Chao (Fig. S2a,b). However, in the late stage of cucumber growth, the Shannon diversity index increased significantly in SBS, SHZ and SM (Fig. S2e). At the same time, the richness index of Chao decreased significantly in SBS (Fig. S2f).

Analysis of the fungal diversity index treatment found that OTU diversity and richness was significantly different in response to each biofertilizer (Fig. S2c,d,g,h). In the early stage of cucumber growth, the Shannon diversity index decreased significantly in SBH, SHZ and SM (Fig. S2c). The richness index of Chao decreased significantly in SBS (Fig. S2d). In the late stage of cucumber growth, the Shannon diversity index increased significantly in S267, SBS, SBH and SM (Fig. S2g). We observed no significant differences in the richness index of Chao (Fig. S2h).

#### Fungal hierarchical cluster analysis at the OTU level

In the early stage of cucumber growth in soil, the taxonomic structure of the microbial community was similar in SBH, S267, SM and SHZ to the control (Fig. S4a). However, we observed that SBS significantly changed the fungal community. In the late stage of cucumber growth, microbial taxa in the SM and the control samples were genetically close compared with the other biofertilizer treatments, which indicated that the soil fungal taxonomic structure was changed more in the other biofertilizer treatments than in SM (Fig. S4c).

In the early stage of cucumber growth in substrate, the control samples in the fungal community were clustered together and well-separated taxonomically from the five biofertilizer treatments (Fig. S4b). However, the five treatments and control were divided into one branch for USCK and the other branch comprising US267, USBH, USBS, USM and USHZ. These results indicated that the five treatments had significantly changed the fungal taxonomic composition.

In the late stage of cucumber growth, however, the five treatments and control were divided into a branch for USCK, USBS, and USBH and another branch comprising US267, USM and USHZ (Fig. S4d). The structure of the microbial community was similar in USBS and the control. The results indicated that US267, USM and USHZ had significantly changed the taxonomic fungal composition in the community.

#### Analysis of differences in fungal genera dominance

**Figure 5:**
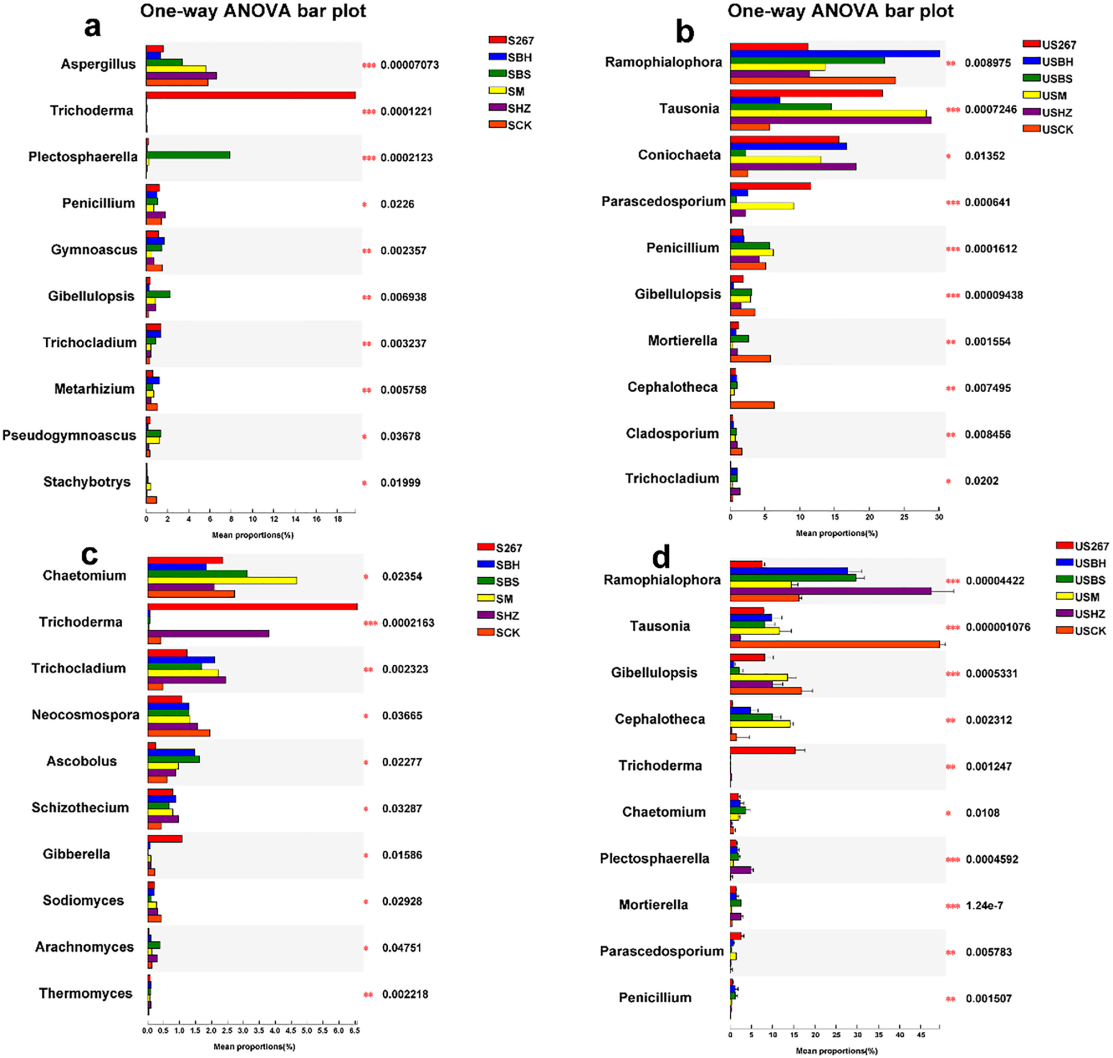
Relative abundance of fungal genera detected in soil and substrate after different biofertilizer treatments in soil (**a** early samples; **c** late samples) and substrate (**b** early samples; **d** late samples). The number of asterisks indicates significant differences between treatments according to a one-way ANOVA and FDR (False Discovery Rate) adjustment (*0.01 < P ≤ 0.05; **0.001 < P ≤ 0.01; ***P ≤ 0.001). S267 = *Trichoderma Strain 2*67 added to soil; SBH = *Bacillus subtilis* and *T. harzianum* biofertilizers added to soil; SBS = *B. subtilis* biofertilizer added to the soil; SM = Compound biofertilizer added to soil; SHZ = *T. harzianum* biofertilizer added to soil; SCK = Untreated soil. US267 = *T.*267 biofertilizer added to substrate; USBH = *B. subtilis* and *T. harzianum* biofertilizers added to substrate; USBS = *B. subtilis* biofertilizer added to substrate; USM = Compound biofertilizer added to substrate; USHZ = *T. harzianum* biofertilizer added to substrate; USCK = Untreated substrate.

We observed significant differences in the relative abundance of fungal genera in soil after microbial fertilizers were applied (Fig. 5a,c). In the early stage of cucumber growth, *Trichoderma* was second only to *Aspergillus* in dominance (Fig. 5a). The relative abundance of *Plectosphaerella* increased significantly in SBS. The relative abundance of *Trichocladium* increased significantly in response to all biofertilizers, whereas *Stachybotrys* decreased significantly. In the late stage of cucumber growth, *Trichoderma* was second only to *Chaetomium* in fungal dominance (Fig. 5c). The relative abundance of *Chaetomium* increased significantly in SM; *Trichoderma* increased significantly in S267 and SHZ; and *Trichocladium* increased significantly SHZ, SM and SBH. In general, the relative abundance of *Neocosmospora* and *Schizothecium* decreased significantly when exposed to all the biofertilizer treatments.

We also observed significant differences in the relative abundance of fungal genera after microbial fertilizer were applied (Fig. 5b,d). In the early stage of cucumber growth, the relative abundance of *Parascedosporium* increased significantly in US267 and USM (Fig. 5b). The relative abundance of *Mortierella* and *Cephalotheca* decreased significantly compared to CK treatment. In the late stage of cucumber growth, the relative abundance of *Ramophialophora* increased significantly in USBH, USBS and USHZ (Fig. 5d). The relative abundance of *Tausonia* decreased significantly in USHZ. The relative abundance of *Gibellulopsis* decreased significantly in USBS and USBH. The relative abundance of *Chaetomium* increased significantly compared with the control in US267, USBH, USBS and USM. In the early and late stage of cucumber, *Tausonia* was second only to *Ramophialophora* in dominance.

**Figure 6:**
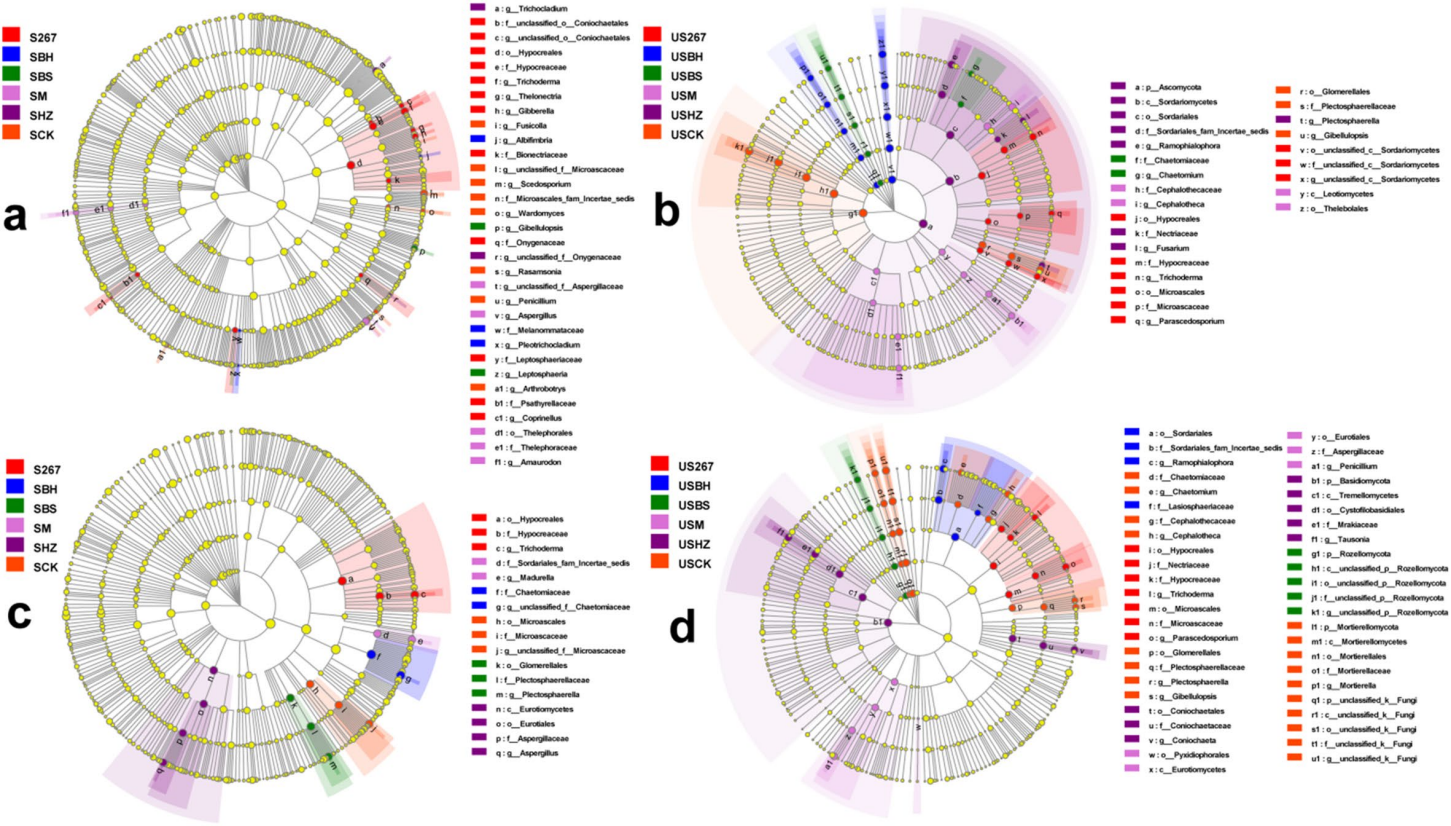
LEfSe cladogram analysis of the species differential abundance in soil (**a** early samples; **c** late samples) and substrate (**b** early samples; **d** late samples) in fungal communities after different biofertilizer treatments. S267 = *Trichoderma Strain 2*67 added to soil; SBH = *Bacillus subtilis* and *T. harzianum* biofertilizers added to soil; SBS = *B. subtilis* biofertilizer added to the soil; SM = Compound biofertilizer added to soil; SHZ = *T. harzianum* biofertilizer added to soil; SCK = Untreated soil. US267 = *T.*267 biofertilizer added to substrate; USBH = *B. subtilis* and *T. harzianum* biofertilizers added to substrate; USBS = *B. subtilis* biofertilizer added to substrate; USM = Compound biofertilizer added to substrate; USHZ = *T. harzianum* biofertilizer added to substrate; USCK = Untreated substrate.

LEfSe analysis identified the biomarkers that caused significant differences in control (P < 0.05, a, c, LDA = 3; b, d, LDA = 4.0; Fig. 6a,b,c,d). We observed that the analyses screened out major taxa at the fungal genus level. In the early stage of cucumber growth in soil, 4, 1, 2, 2, 1 and 3 biomarkers were found in S267, SBH, SBS, SM, SHZ and SCK, respectively. In the late stage of cucumber growth, 5, 3, 10, 3, 7 and 3 biomarkers were found in S267, SBH, SBS, SHZ, SM and SCK, respectively. In the early stage of cucumber growth in substrate, 2, 2, 2, 3 and 2 biomarkers were found in US267, USBS, USM, USHZ and USCK, respectively. In the late stage of cucumber growth, 2, 1, 1, 2 and 5 biomarkers were found in US267, USBH, USM, USHZ and USCK, respectively.

A large LDA score indicates a greater influence of species abundance on the difference effect. We observed that g_*Trichoderma* had larger LDA values in S267 and US267, which indicated that g_*Trichoderma* responded strongly to S267 and US267.

## Discussions

We observed that microbial fertilizers applied to soil or substrate in a greenhouse increased cucumber quality, yield and the abundance of bacterial and fungal genera involved in the control of soil-borne pathogens.

We observed that some of the biofertilizers that we tested increased the growth of cucumbers grown in soil or substrate. Within 10 weeks of applying them, some treatments such as S267 and SHZ significantly increased cucumber plant height and stem diameter. US267, USM and USHZ significantly increased the stem diameter and plant height of substrate-grown cucumbers. Our findings were consistent with the results of Selvakumar^34^ who observed that microbial fertilizers increased the beneficial microorganisms in the rhizosphere and increased plant growth. *B. subtilis* and *Trichoderma* were reported as two of the most important plant growth-promoting organisms in commercial agriculture that increased plant growth and influenced the taxonomic composition in the rhizosphere^35–38^. Some biofertilizers tested were better at increasing cucumber growth in substrate than others. For example, US267, USM and USHZ significantly improved cucumber growth in all cucumber growth stages. Previous research reported that substrates reduced the production constraints inherent in soil production by reduced the use of water, gas for greenhouse heating and fertilizer^39^. Microorganisms present in microbial fertilizers were reported to promote the transformation of organic nutrients in the substrate; accelerate the absorption, transport, assimilation and accumulation of elements by the plant; improve the plant’s utilization rate of nutrients; and improve the yield and quality of cucumber^40^.

Our research showed biofertilizers significantly reduced *Fusarium* and *Phytophthora*, which was also reported by Sridharan and Suriani^41,42^. We also observed that some microbial biofertilizers significantly reduced fungal soil-borne pathogens, even when the cucumber plants were uprooted. That may have been due the presence of beneficial microorganisms in the biofertilizers that had multiplied rapidly and inhibited the growth of pathogens^43^. We observed that 267, BS and HZ were better at controlling soil-borne pathogens than other biofertilizers, but the efficacy of most of them tested reduced during the late growth stage of cucumber. Conversely, in substrate the efficacy of most biofertilizers we tested increased in the late growth stage, which suggested the microbes in our biofertilizers required sufficient time to colonize the substrate. We noted that the microbes were most likely spread in substrates by water that flowed in one direction. In general, the impact of pathogenic bacteria and fungi under continual cropping was delayed with crops produced on substrates compared to soil^44^.

We observed that the biofertilizers had little effect on soil’s bacterial taxonomic diversity, but changed the population abundance of some bacterial taxa which led to a change in community structure. Previous research reported that organic fertilizers can cause changes in the structure of soil microbial communities^45^. In contrast to soil, the biofertilizers applied to substrate significantly affected the bacterial taxonomic diversity. We observed that Strain 267’s increase in bacterial diversity was temporary as bacterial populations levels returned to those observed in the control when the cucumber was uprooted.

We observed that the α diversity of the soil’s fungal community in the changed significantly in both the early and late stages of cucumber growth. In the early stage of cucumber growth, there were no significant differences in the diversity index of Shannon and the richness index of Chao compared with the control. In the late stage of cucumber growth, however, the Shannon diversity index increased significantly compared with the control in SBS, SHZ and SM. In addition, we found that the Shannon diversity index decreased significantly in the SBH, SHZ and SM treatments after the third application of biofertilizers. However, the Shannon diversity index increased significantly compared with the control in S267, SBS, SBH and SM at the time the cucumber plants were uprooted.

The taxonomic tree results showed that some biofertilizer treatments significantly changed the taxonomic composition of the soil and substrate’s bacterial and fungal communities.

In general, we observed that different biofertilizers altered the relative abundance of microbes in different ways. We also observed that soil and substrate microbial diversity differed significantly during the seasonal growth of cucumber. Previous research reported that crop growth stages and fertilization application frequency changed the soil microbial diversity^46^. Some research reported that changes in soil temperature and moisture during the season directly or indirectly influenced the soil microbiome^47^.

We observed that biofertilizers added to soil or substrate changed the bacterial community composition. The rhizosphere microbial communities exhibited distinctly different patterns between soil and substrate. Our soil results are consistent with the findings of previous studies that applied functional bacteria or fungi present in biofertilizers to alter the bacterial taxonomic composition of the soil^48^.

We observed that the relative abundance of *Planifilum, Microvirga, Pedomicrobium* and *Haliangium* increased significantly in the soil in response to biofertilizers.

We found that the relative abundance of *Planifilum* increased significantly in response to biofertilizer application. *Planifilum* is a potential source of *sul* genes, which suggests that the application of organic fertilizer may enrich the potential source bacteria for sul genes, such as *Planifilum*, accelerating the transfer of sul genes^49^. *Planifilum* is also an important nitrogen-fixing bacterium that secretes xylanase to break down xylan^50^, which is a type of hemicellulose found in plants.

We observed that the relative abundance of *Microvirga, Pedomicrobium* and *Haliangium* increased significantly. *Microvirga* spp. are thermo-tolerant bacteria. They are metabolically versatile and widely distributed in nature^51^. In addition, *Microvirga* is also reported to be an environmental remediator, as it can remove residual contaminants in soil and water^52^. *Pedomicrobium* is a ubiquitously occurring hyphal-budding organism that can adhere to surfaces and form biofilms in different habitats^53^. *Haliangium* can produce a type of antifungal metabolite, which inhibits the growth of fungi^54^.

We observed that the relative abundance of *Flavobacterium, Sphingomonas* and *Pseudomonas* are algicidal^55^ bacteria that increased significantly in the substrate in response to biofertilizers.

We observed that the relative abundance of *Actinobacteria* increased significantly in USHZ and USM. *Actinobacteria* are reported to decompose organic matter and inhibit pathogens^56,57^.

*Firmicutes* is drought tolerant and resistant to extreme weather events. It is reported to decontaminate and bioremediate soils that are acidified or contaminated by heavy metals^58,59^. *Firmicutes* may also degrade algae^60^.

We observed an increase in *Trichocladium* fungus, which is involved in lignocellulose degradation^61^. *Mortierella*, and *Tausonia* became the dominant fungi in response to biofertilizers in our research. *Mortierella* is a phosphorus-solubilizing fungus that is important in the soil carbon and phosphorus cycle^62^*. Tausonia* is a member of the phylum Basidiomycota that is reported to produce auxin-like compounds that inhibit pathogens^63^, and ligninase capable of degrading lignin-containing wastes^64^.

The relative abundance of *Gibellulopsis* decreased significantly in USBH and USBS. *Gibellulopsis* is a pathogen that can cause plant diseases^65^.

We observed an increase in the abundance of *Ramophialophora* in soil in response to the biofertilizer treatments. At present, little is known about the function of *Ramophialophora* in soil.

The LEfSe diagram showed that the biofertilizers tested significantly influenced the biological grouping of genera of fungi. In particular, *Trichoderma* 267 significantly changed the soil or substrate bacterial and fungal taxonomic composition. Further studies are needed to verify whether that result was related to the stage of cucumber growth or the “colonization intensity” of both organisms in the microbial fertilizers.

We found that some biofertilizer treatments significantly increased cucumber yield, which was consistent with previous research^66^. Strain 267 and *Trichoderma harzianum* significantly increased cucumber yield more than the other biofertilizers tested. Further studies are needed to verify whether that result was related to the stage of cucumber growth or the “colonization intensity” of both organisms in the microbial fertilizers.

Generally, we observed that biofertilizers added to soil or substrate in a greenhouse improved cucumber growth and increased soil-borne pathogen control. Biofertilizers altered the microbial taxonomic structure. As examples, they significantly increased the relative abundance of some beneficial microorganisms, such as *Planifilum, Microvirga, Pedomicrobium,* and *Haliangium* in soil*,* and increased the relative abundance of *Flavobacterium, Sphingomonas* and *Pseudomonas* in substrate. The extent that biofertilizers changed the taxonomic composition of bacteria and fungi depended to some extent on the stage of the cucumber growth, and the timing of the biofertilizer application in those stages. Our results shed light on the complex interactions between microbes and the plant rhizosphere when a plant is grown in soil or substrate.

Biofertilizers applied to soil or substrate can effectively prevent and control soil-borne diseases, optimize the taxonomic structure of microbial communities, and create a relatively healthy microbial ecological environment for the emergence of many beneficial microorganisms. The ecological functions of soil and substrate were enhanced by increases in the relative abundance of beneficial microorganisms.

The substitution of inorganic fertilizer with biofertilizers will reduce the long term detrimental environmental impact of inorganic fertilizers and improve agricultural sustainability. Biofertilizer were effective in substrate, especially at the later stage of cucumber growth. Substrates obtained from sustainable resources and that are recyclable after use can play a significant role in reducing the accumulation of soil-borne diseases that currently limit the expansion of crops that are continually produced on the same soil each year.

## Supplementary Information

Supplementary Information.

## Data Availability

The datasets generated and/or analysed during the current study are available in the NCBI Sequence Read Archive repository, [accession nos. SRP398183, SRP398194].
