# Supplementary material for: Taxonomic response of bacterial and fungal populations to biofertilizers applied to soil or substrate in greenhouse-grown cucumber
Source: Sci Rep. 2022 Nov 2;12:18522. doi: 10.1038/s41598-022-22673-4 (PMC9630312; doi:10.1038/s41598-022-22673-4)
Supplement: Supplementary file 1 — Supplementary Information. [file 41598_2022_22673_MOESM1_ESM.docx]

Supplementary Material


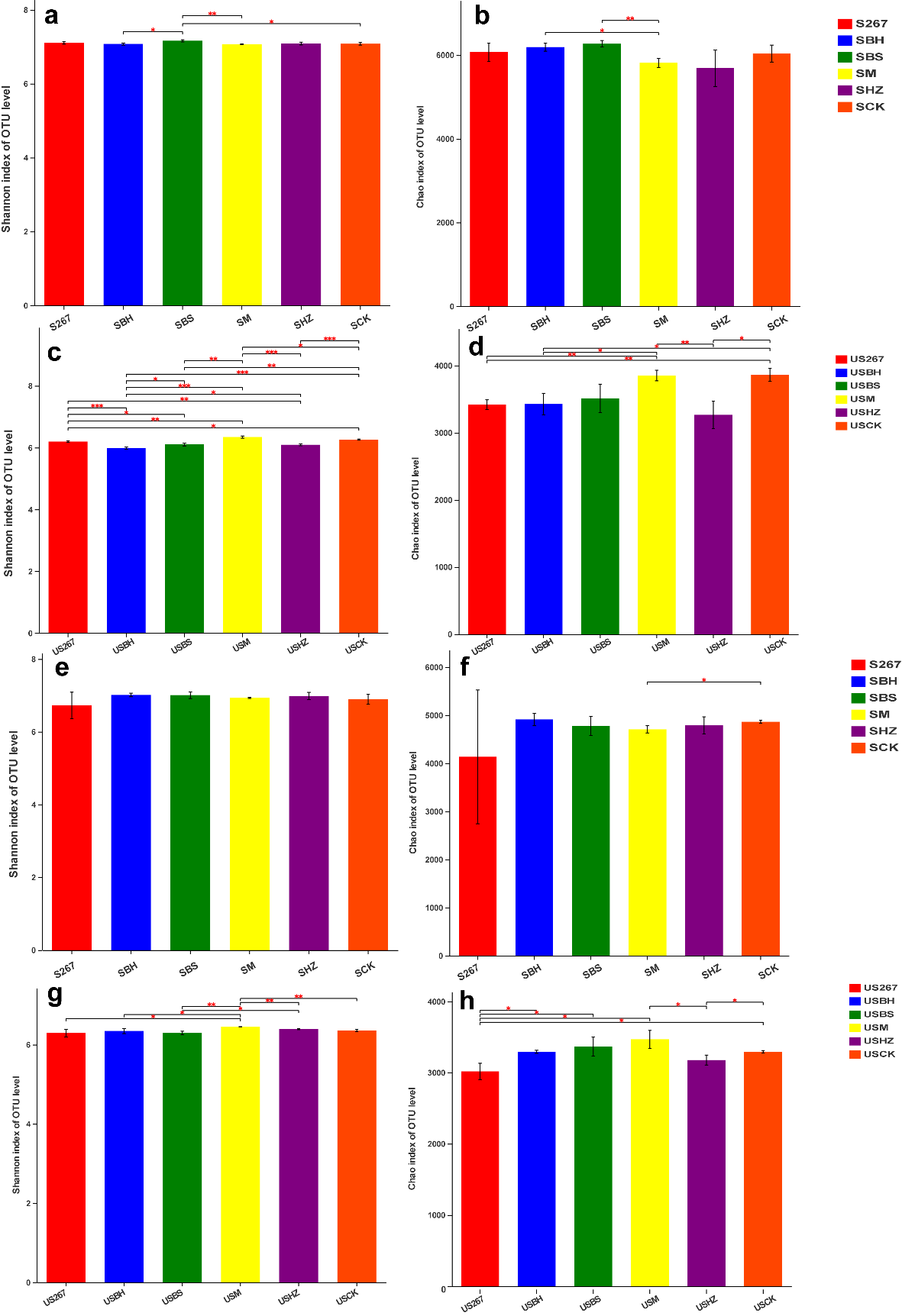


**Fig. S1 The effect of different** **biofertilizers on the alpha diversity of bacterial communities at the OTU level in soil (ab: early samples; ef: late samples) and substrate (cd: early samples; gh: late samples).** The student’s t-test was used to determine differences in alpha diversity between different treatments. Statistical differences between the two groups are marked with an asterisk (* 0.01 < P ≤ 0.05; ** 0.001 < P ≤ 0.01; ***P ≤ 0.001). S267=*Trichoderma Strain 2*67 added to soil; SBH=*Bacillus subtilis* and *T. harzianum* biofertilizers added to soil; SBS=*B. subtilis* biofertilizer added to the soil; SM=Compound biofertilizer added to soil; SHZ=*T. harzianum* biofertilizer added to soil; SCK=Untreated soil. US267=*T.*267 biofertilizer added to substrate; USBH=*B. subtilis* and *T. harzianum* biofertilizers added to substrate; USBS=*B. subtilis* biofertilizer added to substrate; USM=Compound biofertilizer added to substrate; USHZ=*T. harzianum* biofertilizer added to substrate; USCK=Untreated substrate.


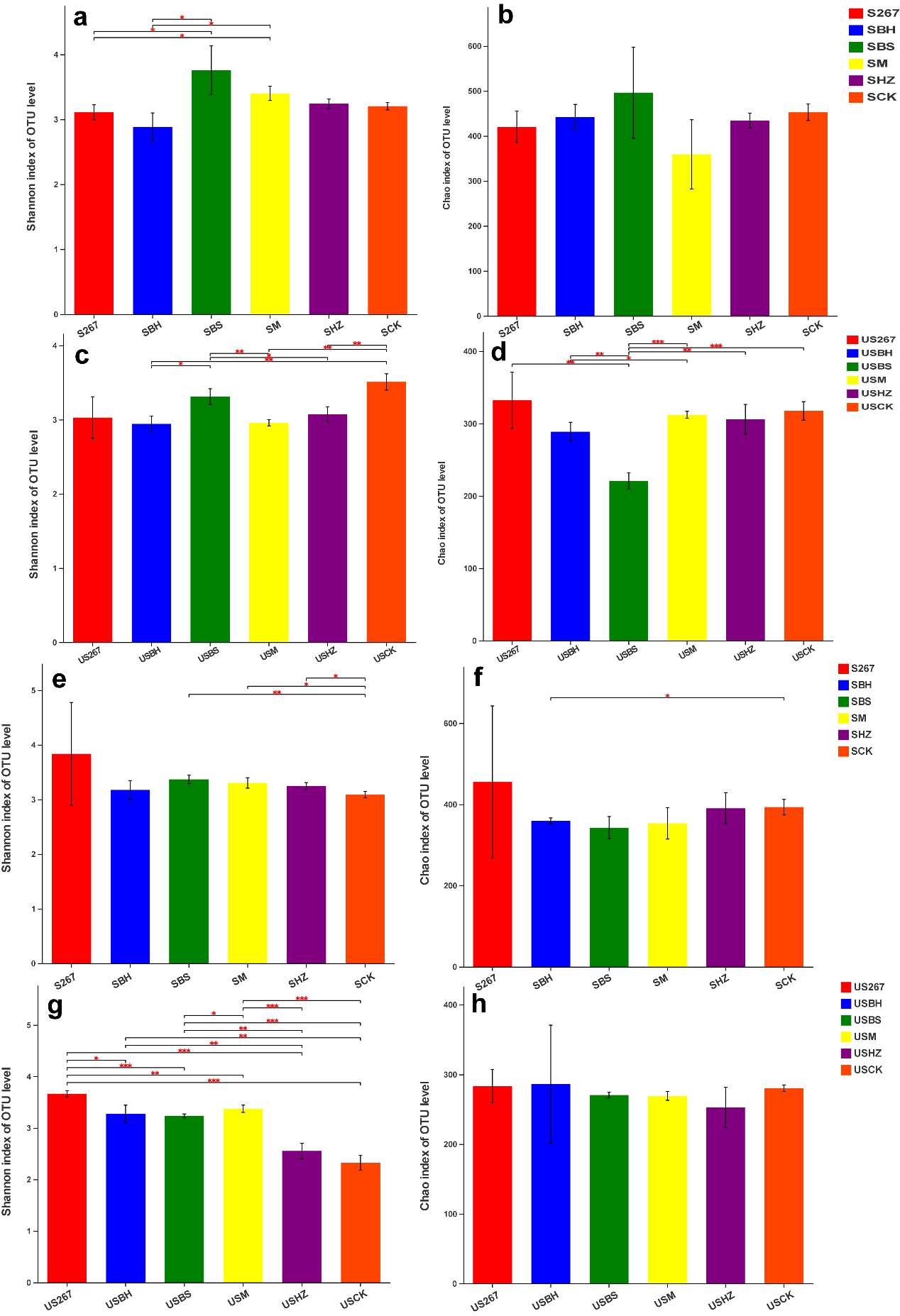


**Fig. S2 The effect of different biofertilizers on the alpha diversity of fungal communities at the OTU level in soil (ab: early samples; ef: late samples) and substrate (cd: early samples; gh: late samples).** The student’s t-test was used to determine differences in alpha diversity between different treatments. Statistical differences between the two groups are marked with an asterisk (* 0.01 < P ≤ 0.05; ** 0.001 < P ≤ 0.01; ***P ≤ 0.001). S267=*Trichoderma Strain 2*67 added to soil; SBH=*Bacillus subtilis* and *T. harzianum* biofertilizers added to soil; SBS=*B. subtilis* biofertilizer added to the soil; SM=Compound biofertilizer added to soil; SHZ=*T. harzianum* biofertilizer added to soil; SCK=Untreated soil. US267=*T.*267 biofertilizer added to substrate; USBH=*B. subtilis* and *T. harzianum* biofertilizers added to substrate; USBS=*B. subtilis* biofertilizer added to substrate; USM=Compound biofertilizer added to substrate; USHZ=*T. harzianum* biofertilizer added to substrate; USCK=Untreated substrate.


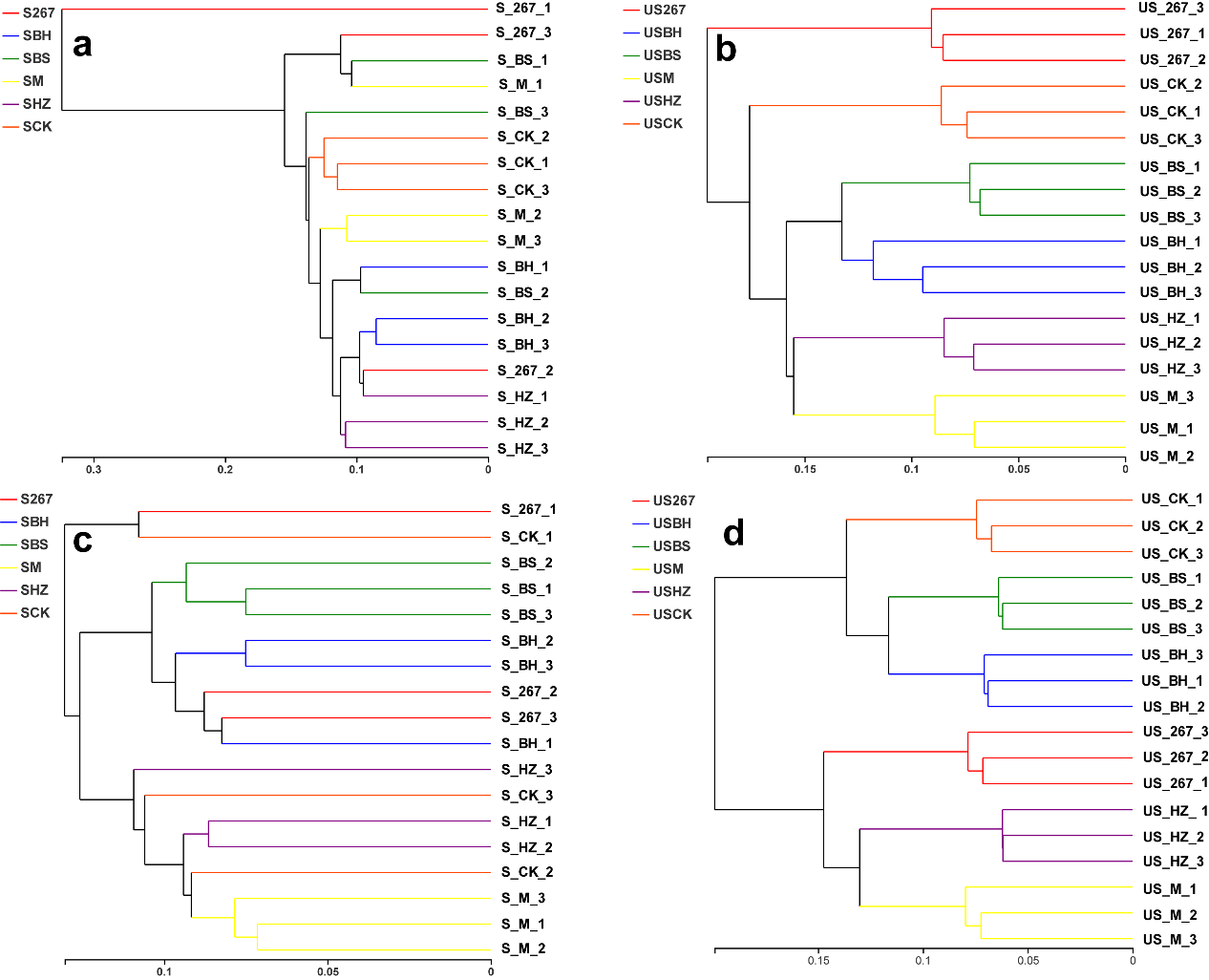


**Fig. S3 Bacterial hierarchical cluster analysis (based on Bray-Curtis method) in soil (a: early samples; c: late samples) and substrate (b: early samples; d: late samples) after different biofertilizer treatments.** S267=*Trichoderma Strain 2*67 added to soil; SBH=*Bacillus subtilis* and *T. harzianum* biofertilizers added to soil; SBS=*B. subtilis* biofertilizer added to the soil; SM=Compound biofertilizer added to soil; SHZ=*T. harzianum* biofertilizer added to soil; SCK=Untreated soil. US267=*T.*267 biofertilizer added to substrate; USBH=*B. subtilis* and *T. harzianum* biofertilizers added to substrate; USBS=*B. subtilis* biofertilizer added to substrate; USM=Compound biofertilizer added to substrate; USHZ=*T. harzianum* biofertilizer added to substrate; USCK=Untreated substrate.


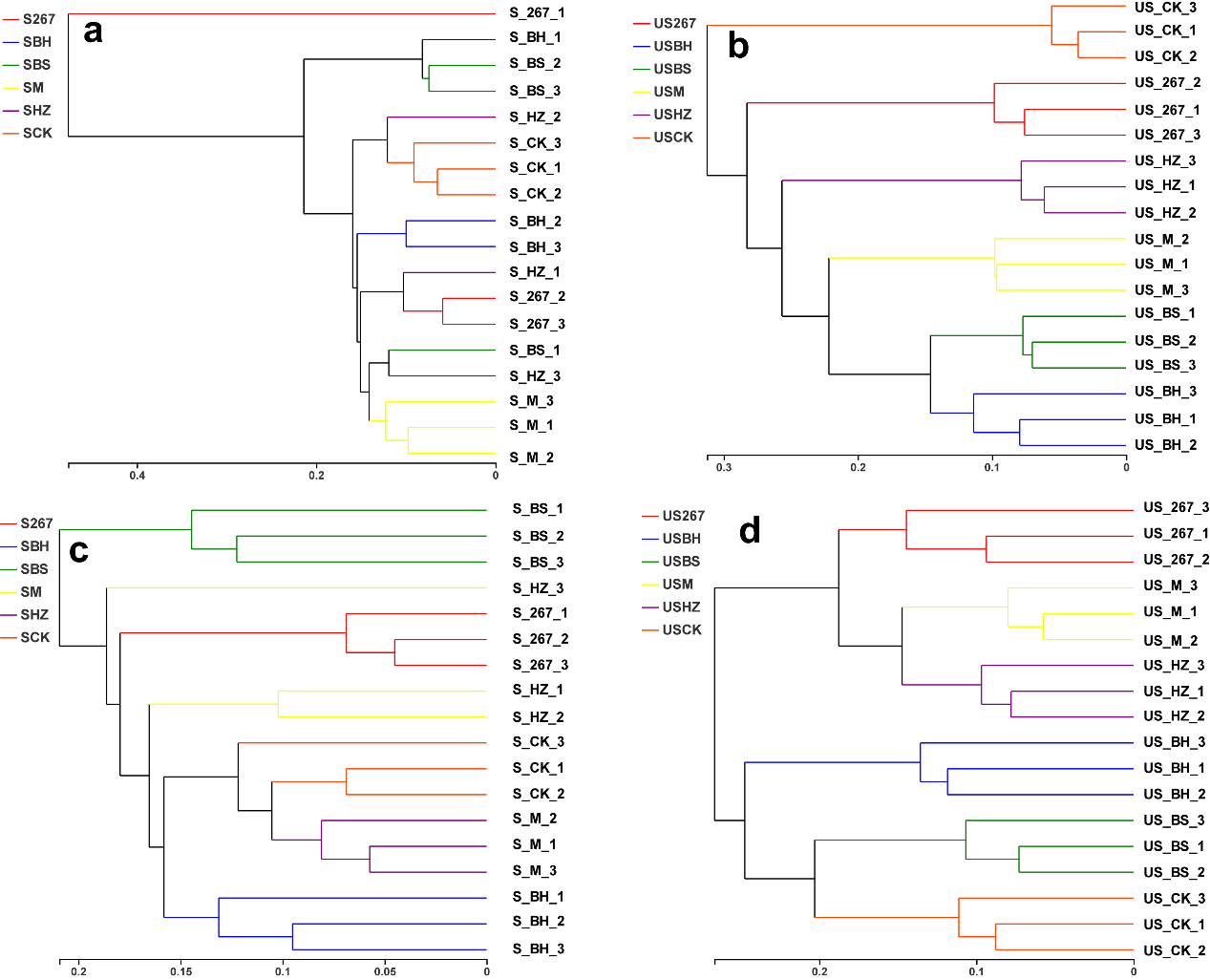


**Fig. S4 Fungal hierarchical cluster analysis (based on Bray-Curtis method) in soil (a: early samples; c: late samples) and substrate (b: early samples; d: late samples) after different biofertilizer treatments.** S267=*Trichoderma Strain 2*67 added to soil; SBH=*Bacillus subtilis* and *T. harzianum* biofertilizers added to soil; SBS=*B. subtilis* biofertilizer added to the soil; SM=Compound biofertilizer added to soil; SHZ=*T. harzianum* biofertilizer added to soil; SCK=Untreated soil. US267=*T.*267 biofertilizer added to substrate; USBH=*B. subtilis* and *T. harzianum* biofertilizers added to substrate; USBS=*B. subtilis* biofertilizer added to substrate; USM=Compound biofertilizer added to substrate; USHZ=*T. harzianum* biofertilizer added to substrate; USCK=Untreated substrate.

**Table S1** **Main physicochemical characteristics of soil and substrate**

| Physicochemical characteristics | NH_4_^+^-N | NO_3_^-^-N | Available phosphorus(mg/kg) | Available potassium(mg/kg) | Organic matter | pH | Electrical conductivity |
| --- | --- | --- | --- | --- | --- | --- | --- |
|  | (mg/kg) | (mg/kg) |  |  | (g/kg) | (1:2.5) | (μs/cm) |
| soil | 0.50 | 709.54 | 324.67 | 894 | 28.93 | 7.30 | 870.00 |
| substrate | 1.27 | 835.98 | 387.81 | 987 | 43.50 | 5.78 | 921.20 |
